# EGFR bypass activation mediates acquired resistance to regorafenib in hepatocellular carcinoma

**DOI:** 10.3389/fmed.2024.1464610

**Published:** 2024-11-13

**Authors:** Lili Hu, Weiwei Shi, Kua Liu, Ding Ma, Qilei Xin, Zhongxia Wang, Yin Cao, Guang Zhang

**Affiliations:** ^1^Division of Hepatobiliary and Transplantation Surgery, Department of General Surgery, Nanjing Drum Tower Hospital, The Affiliated Hospital of Medical School, Nanjing University, Nanjing, China; ^2^Department of Oncological Surgery, The First People’s Hospital of Lianyungang, The First Affiliated Hospital of Kangda College of Nanjing Medical University, Lianyungang, China; ^3^Department of Obstetrics and Gynecology, School of Medicine, Zhongda Hospital, Southeast University, Nanjing, China; ^4^Jiangsu Key Laboratory of Molecular Medicine, Medical School, National Institute of Healthcare Data Science at Nanjing University, Nanjing University, Nanjing, China; ^5^Department of Gastroenterology, Third Xiangya Hospital, Central South University, Changsha, China; ^6^Jinan Microecological Biomedicine Shandong Laboratory, Jinan, China

**Keywords:** regorafenib, acquired resistance, EGFR, bypass activation, combination therapy, HCC

## Abstract

**Background:**

Regorafenib, a tyrosine kinase inhibitor (TKI), is used in the treatment of unresectable hepatocellular carcinoma (HCC). However, the occurrence of acquired resistance limits its antitumor efficacy. While multiple studies have highlighted the crucial role of bypass activation in acquired TKI resistance, few have focused on bypass activation in regorafenib resistance in HCC.

**Methods:**

High-throughput proteomics was used to identify differential proteins associated with bypass activation between acquired regorafenib-resistant cells and parental cells. The ability of epidermal growth factor receptor (EGFR) bypass inhibition to reverse resistance was evaluated both *in vitro* and *in vivo* using direct microscopic observation, the CCK-8 assay, colony formation assay, Annexin V-FITC/propidium iodide double staining, cell cycle analysis, western blotting, and a xenograft model.

**Results:**

The expression of EGFR, a member of the receptor tyrosine kinase (RTK) family, was significantly increased in acquired regorafenib-resistant HCC cells compared with parental cells. Pharmacological inhibition of EGFR with gefitinib restored the sensitivity of regorafenib-resistant HCC cells to regorafenib. In a xenograft mouse model, gefitinib sensitized resistant tumors to regorafenib. Additionally, levels of RAS, RAF, and P-ERK1/2, components of the downstream EGFR signaling pathway, were positively associated with EGFR expression.

**Conclusion:**

EGFR overexpression promotes acquired resistance to regorafenib through RAS/RAF/ERK bypass activation in HCC. Inhibition of EGFR restores sensitivity to regorafenib, and the combination of gefitinib and regorafenib demonstrates significant antitumor efficacy both *in vivo* and *in vitro*. These findings suggest that this combination could be a potential strategy for patients with advanced HCC.

## 1 Introduction

Based on the global cancer statistics of 2020, primary liver cancer was the sixth most commonly diagnosed cancer and the third leading cause of cancer-related death worldwide (1). Hepatocellular carcinoma (HCC) accounts for nearly 90% of all primary liver cancers (2). However, the majority of patients with HCC are diagnosed at an advanced stage, by which time they have lost the opportunity for potential curative treatments such as surgical resection or liver transplantation (3). Therefore, these patients primarily rely on systemic therapies, particularly targeted therapies (4).

Regorafenib, approved by the Food and Drug Administration (FDA) in 2017, is a second-line treatment for patients with advanced HCC (5). This drug is an oral tyrosine kinase inhibitor (TKI) that targets multiple receptor tyrosine kinases (RTKs), such as angiogenic RTKs (VEGFR-1, -2, and -3 and TIE-2), oncogenic RTKs (c-KIT and RET), stromal RTKs (PDGFR-β and FGFR1), and intracellular signaling kinases (c-RAF/RAF-1, BRAF, and BRAFV600E) (6). A clinical study demonstrated that regorafenib was the first drug to show survival benefits in patients with unresectable HCC following sorafenib failure (7). Unfortunately, most patients with HCC eventually develop acquired resistance to regorafenib after a period of treatment (8), resulting in treatment failure and tumor progression (9). Therefore, it is essential to investigate the underlying mechanisms of this resistance and explore potential strategies to overcome it.

To date, few studies have investigated acquired resistance to regorafenib in HCC. Studies on other TKIs have shown that activation of bypass signaling is a nearly universal mechanism of acquired resistance across multiple cancer types (10). Bypass activation occurs when nontargeted RTKs are overexpressed or alternative downstream compounds are abnormally activated, reducing TKI inhibition efficacy (11). For example, the downstream signaling pathways of epidermal growth factor receptor (EGFR), such as Akt or ERK, were aberrantly activated, promoting tumor cell proliferation and leading to EGFR-TKI afatinib resistance in head and neck squamous cell carcinoma (12). Increased activation of PDGFR-β and IGF-IR is frequently observed in patients with melanoma undergoing TKI treatment (13). In breast carcinoma, JNK bypass activation modulates acquired resistance to the HER2-TKI lapatinib (14). Given the prevalence of bypass activation, combining TKIs with inhibitors targeting bypass signaling has become a popular regimen for managing acquired TKI resistance (15). The key to combination therapy lies in the accurate identification of aberrantly activated bypass signaling. However, whether bypass activation is involved in acquired regorafenib resistance in HCC and which specific bypass signaling pathway is activated remains unclear.

In this study, we established regorafenib-resistant HCC cells using a stepwise dose-escalation method and identified aberrantly activated pathways through proteomic analysis. Moreover, combination therapy was used to assess whether inhibition of bypass signaling could reverse regorafenib resistance both *in vitro* and *in vivo*. This study aimed to provide an experimental foundation for the clinical therapy of regorafenib resistance in HCC.

## 2 Materials and methods

### 2.1 Cell culture

The human HCC cell lines, SMMC-7721 and MHCC97H, were purchased from the Cell Bank of the Chinese Academy of Sciences (Shanghai, China). The cells were cultured in Dulbecco’s modified Eagle’s medium (DMEM) supplemented with 10% fetal bovine serum, 100 units/mL penicillin, and 100 mg/mL streptomycin sulfate. They were maintained in a humidified incubator at 37°C with an atmosphere of 5% CO_2_.

### 2.2 Establishment of regorafenib-resistant HCC cells

Acquired regorafenib-resistant HCC cells (97H-R and 7721-R) were established by treating MHCC97H and SMMC-7721 cells, respectively, with gradually increasing concentrations of regorafenib for over 6 months, as described in our previous studies (16).

### 2.3 CCK-8 assay

Cells (10,000 cells/well) were seeded into 96-well plates and cultured for 24 h. CCK-8 solution (10 μL/well; Selleck, Wuhan, China) was added, and the plates were incubated for 1–2 h. Then, the absorbance at 450 nm was measured using a microplate reader (Bio-Rad, CA, United States), and cell viability was determined.

### 2.4 Cell cycle assay

Cells (1 × 10^6^ cells/well) were harvested after various treatments and fixed in 70% ethanol at −20°C for 2 h. After two washes with PBS and centrifugation, the cells were stained with propidium iodide (PI; 10 μg/mL) and RNase A (100 μg/mL) at room temperature for 30 min, followed by their detection using a BD FACSCalibur flow cytometer (Becton Dickinson, San Jose, CA, United States). The distribution of cells in different phases of the cell cycle was analyzed using FlowJo software (Tree Star, San Carlos, CA, United States).

### 2.5 Annexin V-FITC/PI double staining assay

Cells (1 × 10^6^ cells/well) were collected and resuspended in 500 μL of binding buffer. Then, 5 μL of Annexin V-FITC staining solution and 5 μL of PI staining solution were added to the suspension, followed by incubation for 30 min at room temperature. Fluorescence intensity was measured using a BD FACSCalibur cytometer (Becton Dickinson), and the percentage of apoptotic cells was determined using FlowJo software (Tree Star).

### 2.6 Colony formation assay

Cells (1,000 cells/well) were plated in six-well plates and monitored for 7–14 days to assess colony growth. Afterward, the cells were fixed with 4% paraformaldehyde for 20 min, washed, and incubated with 0.4% crystal violet solution for 30 min. The cells were then washed with PBS, dried, and counted. Colonies containing more than 30 cells were included in the count.

### 2.7 Protein isolation and western blotting

Cells were lysed on ice for 5 min using 150 μL of lysis buffer (Beyotime, Shanghai, China) containing 1% protease inhibitors (Thermo Fisher Scientific, Waltham, MA, United States). The lysates were then harvested and centrifuged at 12,000 × g for 5 min at 4°C. Protein concentrations were determined using a BCA kit (Thermo Fisher Scientific). Equal amounts of protein (20 μg/lane) were separated by sodium dodecyl sulfate-polyacrylamide gel electrophoresis (SDS-PAGE), transferred onto polyvinylidene difluoride (PVDF) membranes, and blocked with 5% nonfat dry milk for 1 h at room temperature. Immunoblotting was performed by incubating the membranes with primary antibodies overnight at 4°C, followed by incubation with secondary antibodies the next day. Protein signals were detected using an enhanced chemiluminescence reagent (EMD Millipore, Billerica, MA, United States).

### 2.8 Bioinformatic analysis

Differentially expressed proteins (DEPs) were identified using the following reference thresholds: a fold change of 1.2 and a *p*-value of <0.05. Gene Ontology (GO) analysis was performed via the GO Database^1^ using Blast2GO (version 3.3.5). The GO functions of DEPs, including biological process (BP), molecular function (MF), and cellular component (CC), were analyzed.

### 2.9 HCC orthotopic xenograft assay

The study protocol was approved by the Institutional Ethics Committee of the Affiliated Drum Tower Hospital, Nanjing Medical University. We sourced six-week-old male BALB/c nude mice from the Model Animal Research Center at Nanjing University and maintained them in accordance with the institution’s guidelines. Two nude mice were subcutaneously injected with 1 × 10^7^ regorafenib-resistant MHCC-97H cells in 100 μL of PBS. Once the tumors reached a volume of 100 mm^3^, they were excised, dissected into 1 mm^3^ segments, and implanted subcutaneously on the dorsal side near the right armpit of the mice. The mice were then randomly assigned to four groups and received oral treatments of either vehicle control, regorafenib, gefitinib, or a combination of regorafenib and gefitinib. The dosages were 20 mg/kg for regorafenib and 50 mg/kg for gefitinib, administered orally once daily for an initial period of 21 days. Body weight and tumor volume were monitored every 2 days. After 4 weeks of treatment, the mice were euthanized, and the tumors were collected for further analysis.

### 2.10 Hematoxylin and eosin (H&E) staining

Tissues fixed with 4% paraformaldehyde were embedded in paraffin and sectioned into 5 μm thick slices. The tissue sections were deparaffinized in xylene, rehydrated through a graded series of ethanol, and washed in PBS. They were then stained with hematoxylin, agitated for 30 s, and rinsed in water. Subsequently, they were stained with eosin, agitated for 10–30 s, and rinsed in water. After staining, the sections were dehydrated, mounted, and covered with a cover slip. Microscopic examination was performed using a microscope (BX50; Olympus, Tokyo, Japan).

### 2.11 Statistical analysis

PSS 19.0 statistical software (IBM Corp., Chicago, IL, United States) was used for data analysis. Data are presented as means ± SD from triplicate experiments. Comparisons between two groups were performed using the Student’s *t*-test. Significance among multiple groups was determined by one-way analysis of variance followed by the Student–Newman–Keuls *post hoc* test. A *p*-value of <0.05 was considered to indicate a statistically significant difference.

## 3 Results

### 3.1 EGFR is overexpressed in regorafenib-resistant HCC cell lines

**Figure 1:**
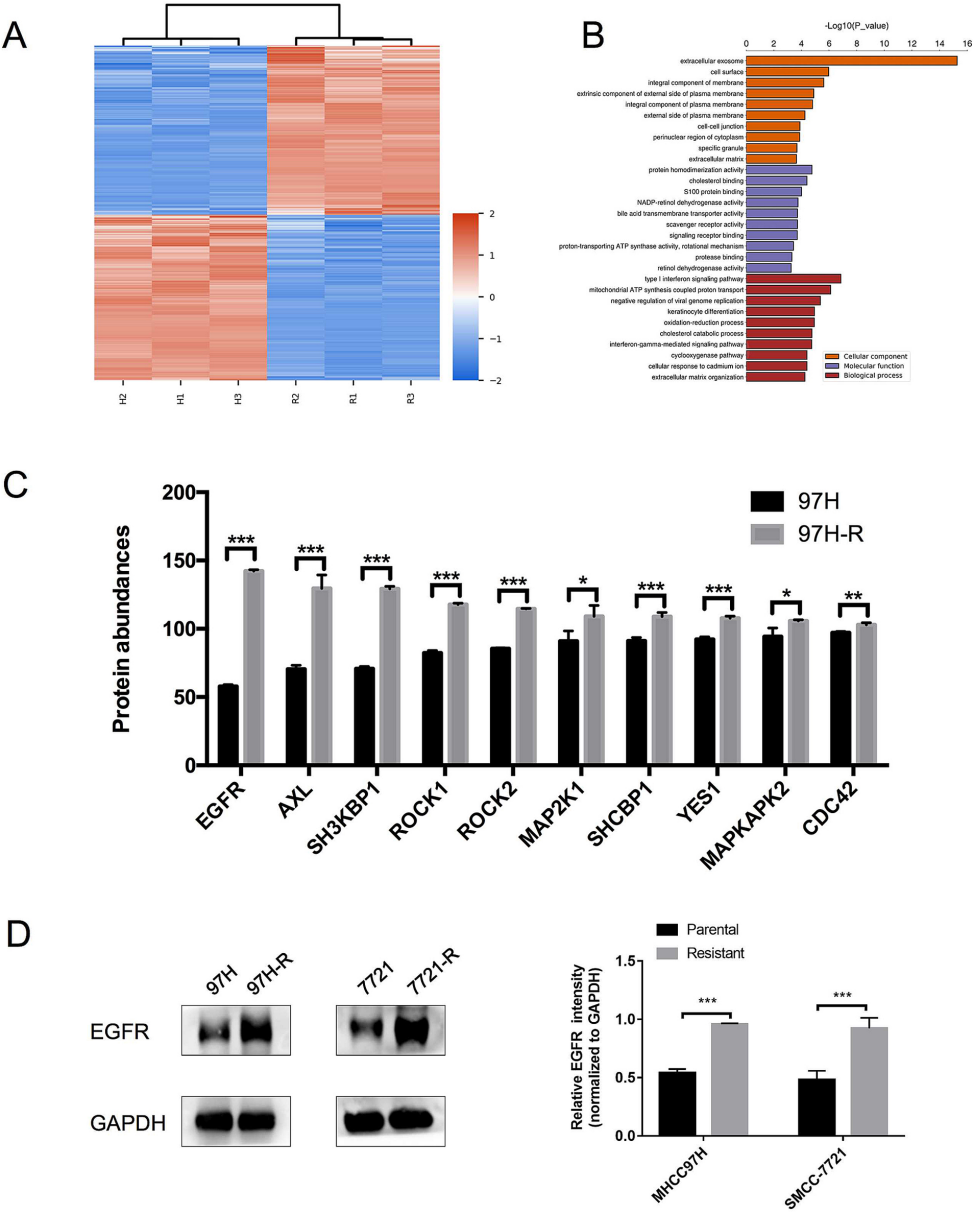
Expression levels of EGFR in regorafenib-resistant HCC cells and parental cells. (A) Volcano plot of DEPs between parental cells and regorafenib-resistant HCC cells. (B) GO enrichment analysis for DEPs. (C) The top 10 proteins associated with RTK pathways overexpressed in 97H-R cells compared with MHCC97H cells. (D) Protein expression levels of EGFR in parental and regorafenib-resistant HCC cells, as measured by western blotting. Results are representative of three independent experiments. Error bars represent the mean ± SD from a representative experiment. ^*^p < 0.05 and ^***^p < 0.001.

Regorafenib-resistant HCC cell lines were established using MHCC97H and SMMC-7721 cell lines through a stepwise dose-escalation method and were designated as 97H-R and 7721-R, respectively. The successful establishment of regorafenib-resistant HCC cells was confirmed using the CCK-8 assay. The IC_50_ values for regorafenib (Table 1) were markedly higher in the resistant cells (97H-R: 16.85 μM; 7721-R: 12.27 μM) compared with the parental cells (97H: 6.266 μM; 7721: 5.431 μM). Based on high-throughput proteomics analysis, we identified DEPs between MHCC97H and 97H-R cells. These DEPs were used to generate a heatmap (Figure 1A), which demonstrated a significant difference in protein expression between the resistant and parental cells. The detailed list of DEPs is provided in Supplementary Table S1. GO enrichment analysis showed that the DEPs were primarily enriched in cell division under the CC subcategory (Figure 1B). EGFR, a key member of the tyrosine kinase receptor family, was the most overexpressed protein associated with RTK pathways. The protein level of EGFR in 97H-R cells was 2.46-fold higher than that in MHCC97H cells (Figure 1C). Western blotting analysis further confirmed that the protein expression of EGFR was significantly upregulated in regorafenib-resistant cells compared with parental cells (*p* < 0.001; Figure 1D). These results indicate that EGFR might mediate regorafenib resistance in HCC.

**Table 1:** IC_50_ values of regorafenib in parental HCC cells and regorafenib-resistant HCC cells.

Cell line	IC_50_ (μM)
97H	5.378
97H-R	16.85
7721	5.431
7721R	12.27

### 3.2 Pharmacological inhibition of EGFR using gefitinib restores the regorafenib sensitivity in regorafenib-resistant HCC cells

**Figure 2:**
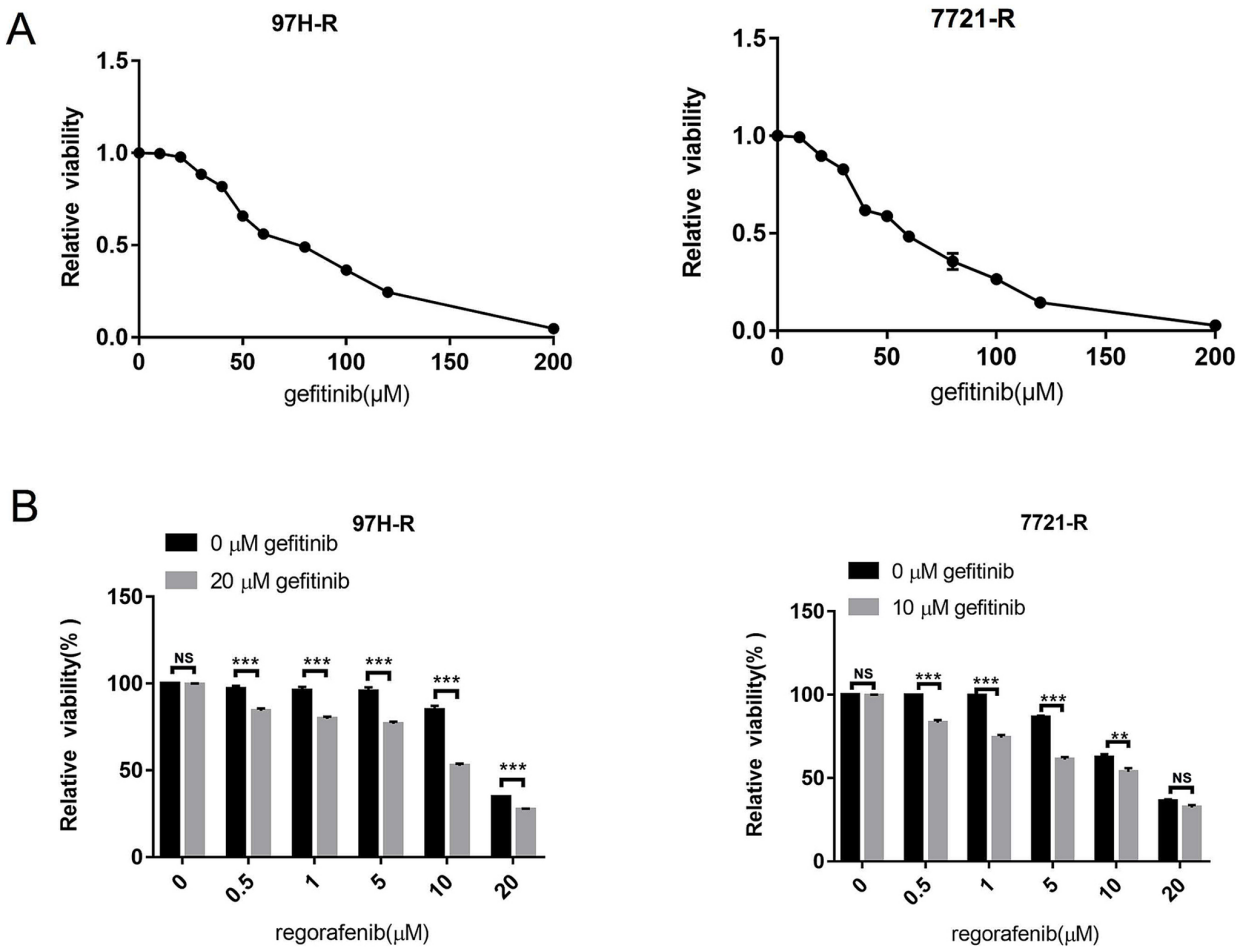
Effect of EGFR inhibition on the reversal of regorafenib resistance. (A) 97H-R cells were treated with 20 μM gefitinib, and 7721-R cells were treated with 10 μM gefitinib for 48 h. The impact of gefitinib on the viability of regorafenib-resistant HCC cells was evaluated using the CCK-8 assay. (B) The viability of regorafenib-resistant HCC cells treated with regorafenib, with or without gefitinib, was measured. The results are representative of three independent experiments. Error bars represent the mean ± SD from a representative experiment. ^**^p < 0.01 and ^***^p < 0.001.

To assess the role of EGFR in regorafenib resistance in HCC, we used gefitinib, a selective inhibitor of EGFR. We first evaluated the effect of gefitinib on the viability of 97H-R and 7721-R cells using the CCK-8 assay. Treatment with gefitinib alone at concentrations up to 20 μM for 48 h had minimal inhibitory effect on the viability of 97H-R cells, while the inhibitory concentration for 7721-R cells was 10 μM (Figure 2A). However, when combined with regorafenib, the viability of 97H-R cells decreased after treatment with 20 μM gefitinib for 48 h (10 μM gefitinib for 7721-R cells; Figure 2B). The IC_50_ values for regorafenib in 97H-R and 7721-R cells also decreased following gefitinib supplementation (Table 2). These results indicate that EGFR inhibition reverses regorafenib resistance in HCC cells.

**Table 2:** IC_50_ values of regorafenib in regorafenib-resistant HCC cells exposed to gefitinib and control group cells.

Cell line	Group	IC_50_ (μM)
97H-R	0 μM gefitinib	15.12
	20 μM gefitinib	6.156
7721-R	0 μM gefitinib	12.31
	10 μM gefitinib	7.142

### 3.3 Combination of gefitinib and regorafenib exhibits strong antitumor potency *in vitro*

**Figure 3:**
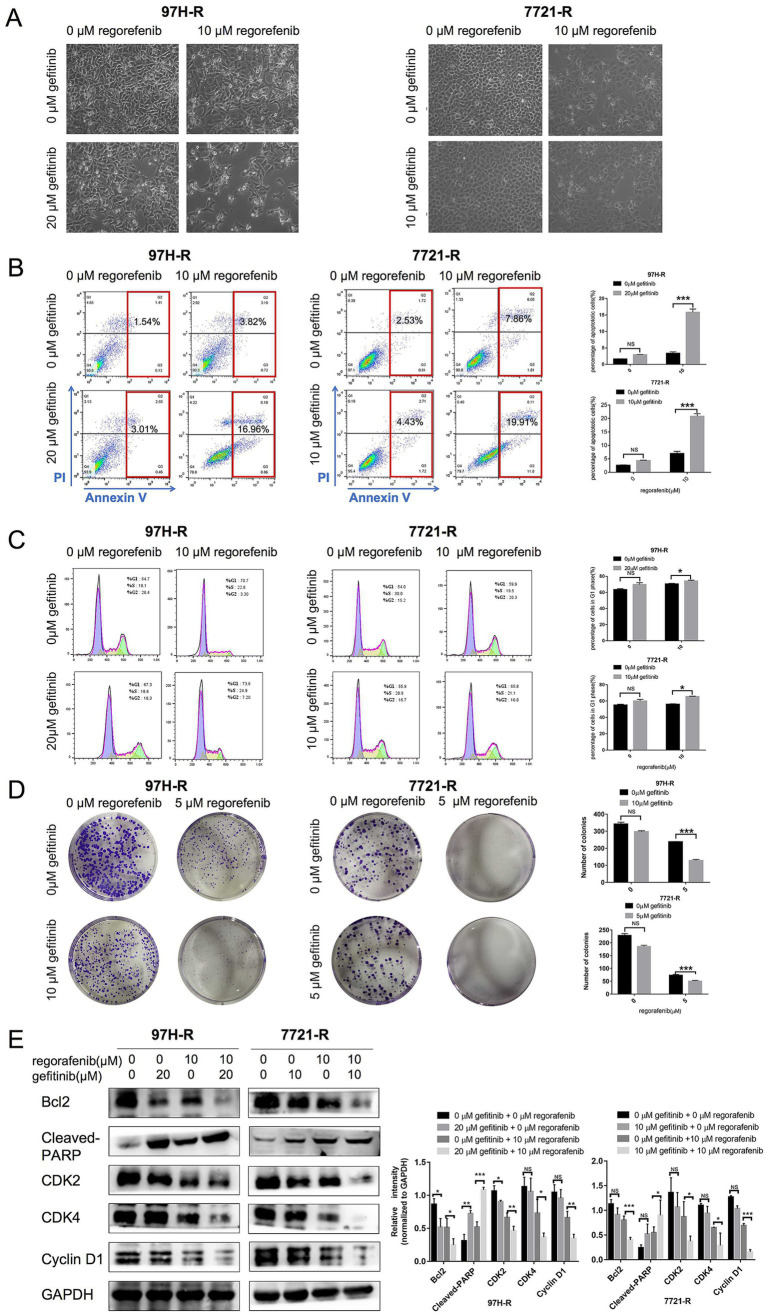
Effect of regorafenib and gefitinib combination treatment on the proliferation and apoptosis of regorafenib-resistant HCC cells. (A) Regorafenib-resistant HCC cells were treated with gefitinib and regorafenib for 48 h. Representative images of cell morphology were captured. Subsequently, (B) the apoptosis rate was measured using the Annexin V-FITC/PI double-staining assay. (C) Cell cycle distribution was analyzed by flow cytometry, and (D) cell proliferation was evaluated using the colony formation assay. (E) The expression levels of Bcl-2, cleaved PARP, cyclin D1, CDK2, and CDK4 were examined by western blotting. The concentrations of regorafenib and gefitinib in all assays for 97H-R cells were 10 and 20 μM for 48 h, respectively, except for the colony formation assay (5 and 10 μM, 10 days). For all assays in 7721-R cells, the concentrations were 10 and 10 μM for 48 h, respectively, except for the colony formation assay (5 and 5 μM, 10 days). The results represent three independent experiments. Error bars indicate the mean ± SD from a representative experiment. ^*^p < 0.05, ^**^p < 0.01, and ^***^p < 0.001.

We further evaluated the antitumor efficacy of the combination treatment with gefitinib and regorafenib *in vitro*. The results showed that both the number and cell state of 97H-R and 7721-R cells were decreased when exposed to the combination of regorafenib and gefitinib, as observed through direct microscopy. In contrast, treatment with either regorafenib or gefitinib alone had minimal impact on the cell number or cell state of these two cell lines (Figure 3A). The proportion of apoptotic cells was significantly higher in the combination treatment group compared with the other groups (97H-R: *p* < 0.001; 7721-R: *p* < 0.001; Figure 3B). Moreover, the combination treatment increased the number of cells arrested in the G1 phase (Figure 3C) and reduced colony formation (Figure 3D) by 97H-R and 7721-R cells. To further confirm the differential effects of gefitinib and regorafenib on cell apoptosis, we measured the protein levels of two apoptotic cascade-related proteins, B-cell leukemia/lymphoma 2 (Bcl2) and cleaved poly ADP-ribose polymerase (PARP). The effects on cell proliferation were verified by examining the expression levels of cyclin D1 and cyclin-dependent kinases 2 and 4 (CDK2 and CDK4). The alterations in the protein levels of Bcl2, cleaved PARP, cyclin D1, CDK2, and CDK4 further confirmed that the combination of gefitinib and regorafenib indeed promoted cell apoptosis and inhibited the proliferation of regorafenib-resistant HCC cells (Figure 3E). These results suggest that inhibition of EGFR may reduce regorafenib resistance in HCC cells.

### 3.4 Gefitinib supplementation reverses regorafenib-resistant HCC tumor growth in a mouse model

**Figure 4:**
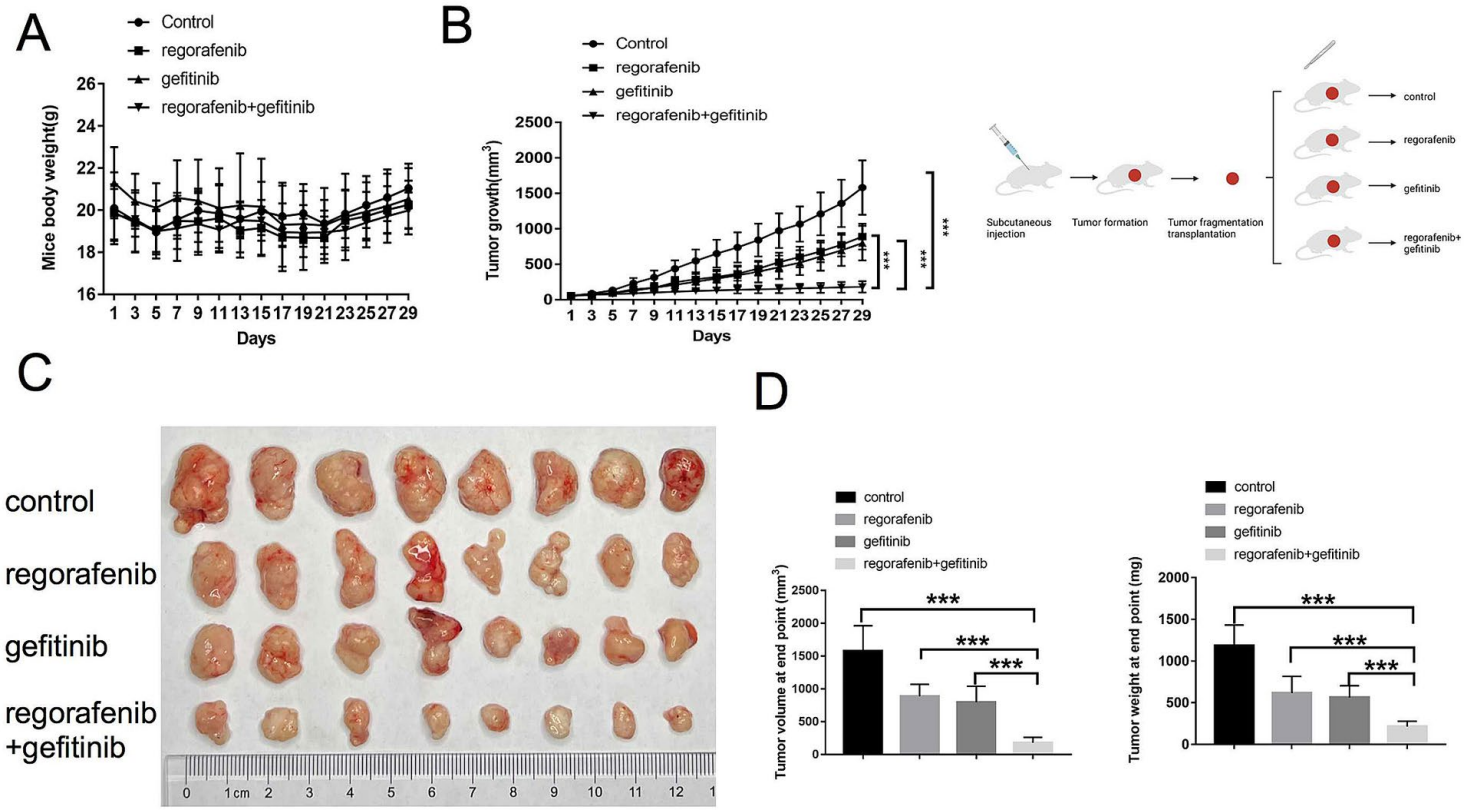
Effects of regorafenib resistance reversal by gefitinib in a xenograft nude mouse model of HCC. (A) Body weight of nude mice during drug treatment. (B) Tumor growth of subcutaneous xenograft tumors in nude mice during drug treatment. (C) Images of tumors harvested from nude mice treated with vehicle, regorafenib, gefitinib, or a combination of regorafenib and gefitinib. (D) Average volume and weight of subcutaneous xenograft tumors harvested from nude mice at the study’s endpoint. Error bars represent the mean ± SD. ^**^p < 0.01 and ^*^p < 0.001.

This study further investigated whether inhibition of EGFR by gefitinib could reverse regorafenib resistance in a nude mouse xenograft model established with 97H-R cells. No significant difference in body weight was observed between the drug-treated mice and the vehicle-treated controls, and no mice died during the treatment, indicating minimal systemic toxicity from the drugs (Figure 4A). Furthermore, H&E staining of lung, kidney, liver and heart tissues indicated barely any difference between the groups (Supplementary Figure S1). As shown in Figures 4B,C, treatment with either regorafenib or gefitinib alone produced a mild tumor inhibitory effect compared with the vehicle control, while the combination of the two drugs significantly inhibited tumor growth. The volume and weight of the tumors at the end confirmed that supplementation with gefitinib sensitized regorafenib-resistant HCC tumors to regorafenib treatment (Figure 4D). These results indicate that combination therapy with gefitinib and regorafenib exerts a strong tumor-inhibitory effect *in vivo*.

### 3.5 EFGR regulates acquired regorafenib resistance in HCC via RAS/RAF/ERK signaling activation

**Figure 5:**
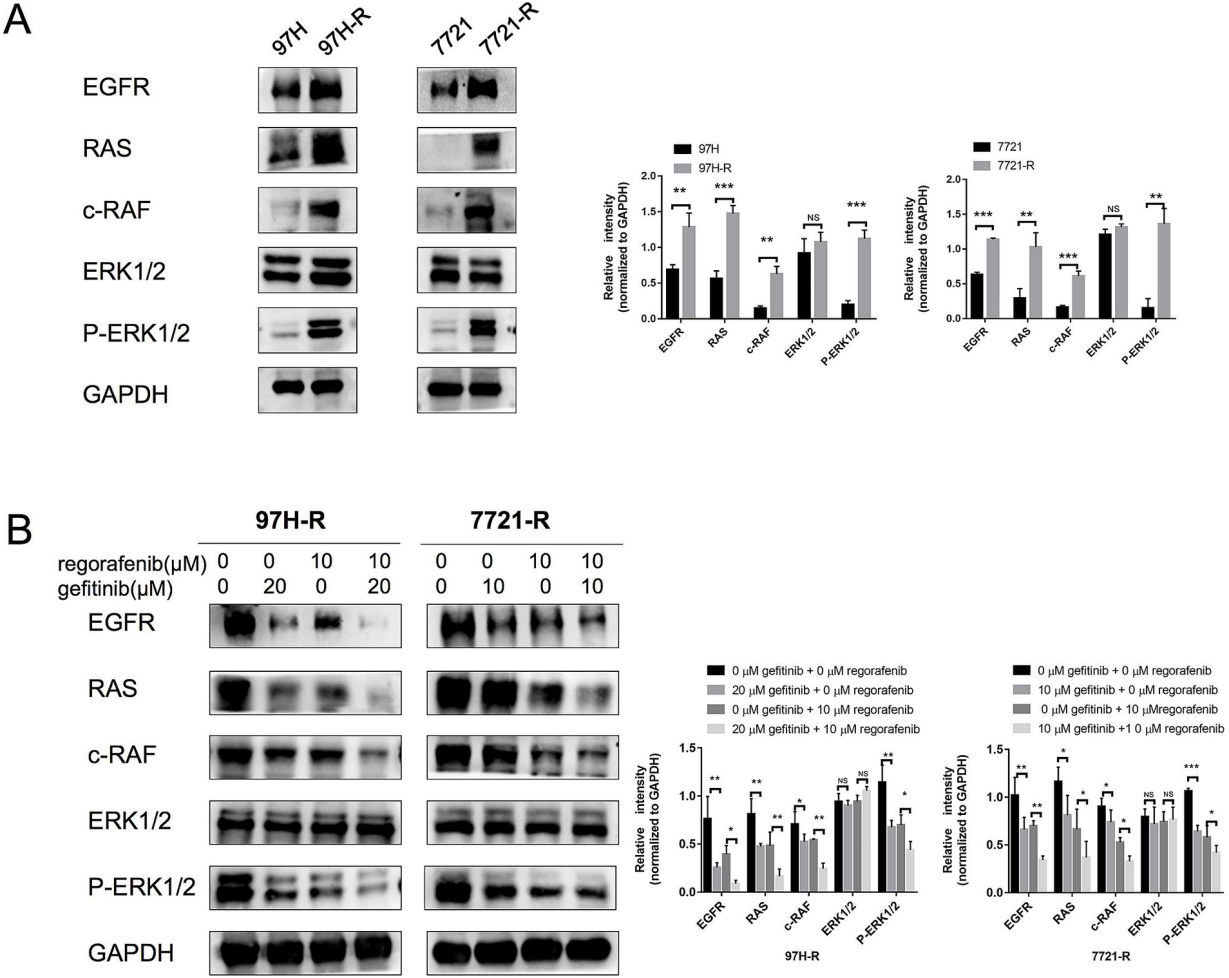
Protein levels of RAS, c-RAF, and ERK1/2 and the phosphorylation level of ERK1/2 in HCC cells. (A) The expression levels of RAS, c-RAF, ERK1/2, and P-ERK1/2 in both parental and regorafenib-resistant HCC cells were examined by western blotting. (B) 97H-R cells were treated with 10 μM regorafenib and 20 μM gefitinib for 48 h, while 7721-R cells were treated with 10 μM regorafenib and 10 μM gefitinib for 48 h. The expression levels of RAS, c-RAF, ERK1/2, and P-ERK1/2 were then examined. Error bars represent the mean ± SD from a representative experiment. ^*^p < 0.05, ^**^p < 0.01, and ^***^p < 0.001.

Although we confirmed that EGFR overexpression promotes acquired regorafenib resistance in HCC, the underlying molecular mechanisms remained unclear. Previous studies have identified the RAS/RAF/ERK signaling as an important downstream pathway of EGFR (17, 18). Therefore, we first measured the protein levels of RAS, c-RAF, and ERK1/2, as well as the phosphorylation of ERK1/2, in regorafenib-resistant cells (97H-R and 7721-R) and their corresponding parental cells (MHCC97H and SMMC-7721). No significant differences in ERK1/2 protein levels were observed between MHCC97H and 97H-R cells; however, the protein levels of RAS, c-RAF, and P-ERK1/2 were markedly higher in 97H-R cells compared with those in MHCC97H cells (Figure 5A). Similar results were obtained for SMMC-7721 and 7721-R cells. Additionally, we assessed the protein levels of RAS, c-RAF, ERK1/2, and P-ERK1/2 following EGFR inhibition by gefitinib in 97H-R and 7721-R cells. EGFR inhibition significantly reduced the elevated expression of RAS, c-RAF, and P-ERK1/2. Furthermore, these protein levels were significantly decreased after combined treatment with regorafenib and gefitinib (Figure 5B). These findings indicate that EGFR overexpression promotes regorafenib resistance by activating the RAS/RAF/ERK signaling pathway.

## 4 Discussion

Acquired resistance is a highly complex challenge in cancer treatment that limits the effectiveness of TKI therapies (19). Multiple mechanisms mediate the acquired resistance to regorafenib in HCC, such as immunosuppression (20), gut microbiome (21), and oxidative stress (22). However, the key factor driving this resistance remains unknown. This study is the first to demonstrate that EGFR plays a crucial role in mediating acquired resistance to regorafenib through activation of the RAS/RAF/ERK pathway in HCC. In addition, gefitinib, a selective inhibitor of EGFR, showed high potential to enhance the sensitivity of regorafenib-resistant HCC cells to regorafenib.

EGFR belongs to the subfamily of RTKs. Ligand binding induces EGFR dimerization, leading to the phosphorylation of intracellular tyrosine residues, which activates downstream pathways that regulate critical BPs, such as cell proliferation, migration, metastasis, and survival (23). Abnormal expression or activation of EGFR and its downstream pathways is common in various cancers, such as gastric cancer (24), colorectal cancer (25), and breast cancer (26). In HCC, high EGFR expression has been associated with poor prognosis (27). Moreover, Jin et al. (28) reported that EGFR activation limits the response of patients with HCC to lenvatinib (another TKI used for HCC therapy) and that EGFR inhibition sensitizes HCC to lenvatinib. However, it remains unclear whether EGFR mediates acquired regorafenib resistance in HCC. Our study is the first to show that EGFR is overexpressed in regorafenib-resistant HCC cells. To further investigate the role of EGFR in the acquired resistance of HCC to regorafenib, we performed various *in vitro* and *in vivo* experiments to determine whether EGFR inhibition by gefitinib could restore regorafenib sensitivity in HCC. We found that the combination of regorafenib with gefitinib significantly inhibited the growth and proliferation of regorafenib-resistant HCC cells and induced cell apoptosis. Upon EGFR inhibition by gefitinib, the IC_50_ of regorafenib for the two types of acquired-resistant cells decreased significantly. Additionally, our experimental results demonstrated that the combination therapy of regorafenib and gefitinib markedly inhibited tumor growth in a xenograft model of regorafenib-resistant HCC cells, compared with treatment with regorafenib or gefitinib alone. Taken together, these findings confirm that EGFR overexpression facilitates regorafenib resistance in HCC. Furthermore, our results suggest that the EGFR inhibitor gefitinib has the potential to reverse regorafenib resistance, offering significant clinical value for the treatment of regorafenib-resistant patients with HCC. Although regorafenib and gefitinib target different tyrosine kinases, there is an overlap in the intracellular signaling pathways they inhibit, such as the RAS/RAF/ERK and PI3K/Akt pathways (29, 30). On the one side, this may contribute to the synergistic antitumor effect of the regorafenib and gefitinib combination in HCC (31). On the other hand, the abnormal activation of these same target cellular pathways has the potential to induce cross-resistance to both drugs (32). In our study, the regorafenib-resistant HCC cells did not develop resistance to gefitinib. One possible explanation is that gefitinib’s inhibitory effect on the RAS/RAF/ERK is stronger than that of regorafenib.

Bypass activation is a critical cause of acquired TKI resistance, which reactivates key downstream signals to promote cancer cell proliferation and/or survival despite the inhibition of the original targets (33). EGFR, a member of the RTK family, is not a target of regorafenib but is overexpressed in regorafenib-resistant HCC cells. We hypothesized that EGFR bypass activation regulates acquired regorafenib resistance in HCC, though the key downstream signal of EGFR remains unknown. This study demonstrates that the RAS/RAF/ERK pathway plays a role in the acquired resistance to regorafenib in HCC. Western blot analysis showed higher levels of RAS, c-RAF, and P-ERK1/2 in regorafenib-resistant cells compared with parental cells. Since inhibition of the RAS/RAF/ERK pathway is a primary antitumor mechanism of regorafenib, EGFR overexpression-mediated activation of the RAS/RAF/ERK pathway counteracts regorafenib’s inhibitory effects, resulting in acquired resistance in HCC. Similar bypass activation phenomena have been observed in acquired erlotinib resistance in non-small cell lung cancer (NSCLC), where AXL, an RTK subfamily member, increased and activated Akt/MAPK downstream signaling, leading to a loss of erlotinib’s effectiveness in blocking the Akt/MAPK pathway (34). Bypass IGF1R/RAS/RAF/ERK activation has also been reported to drive sorafenib resistance in HCC (35). Although we identified EGFR/RAS/RAF/ERK bypass activation as a contributor to acquired regorafenib resistance in HCC, the reasons for EGFR overexpression remain unclear. Further experiments are needed to explore the underlying mechanisms.

## 5 Conclusion

Collectively, our findings suggest that EGFR overexpression mediates acquired resistance to regorafenib in HCC through downstream RAS/RAF/ERK bypass activation. Most notably, the combination of regorafenib with gefitinib inhibits proliferation and promotes apoptosis in regorafenib-resistant HCC cells, indicating that EGFR inhibition by gefitinib has the potential to overcome acquired resistance to regorafenib treatment. Our study provides new insights into therapeutic strategies for patients with advanced HCC exhibiting regorafenib resistance.

## Data Availability

The raw data supporting the conclusions of this article will be made available by the authors, without undue reservation.

## Footnotes

^1^ http://www.geneontology.org

## Supplementary material

SUPPLEMENTARY DATA SHEET 1 DEPs between MHCC97H and 97H-R cells.

SUPPLEMENTARY FIGURE 1 HE staining of lung, kidney, liver and heart.
