# Supplementary figures and images for: EGFR bypass activation mediates acquired resistance to regorafenib in hepatocellular carcinoma
Source: Front Med (Lausanne). 2024 Nov 13;11:1464610. doi: 10.3389/fmed.2024.1464610 (PMC11598357; doi:10.3389/fmed.2024.1464610)

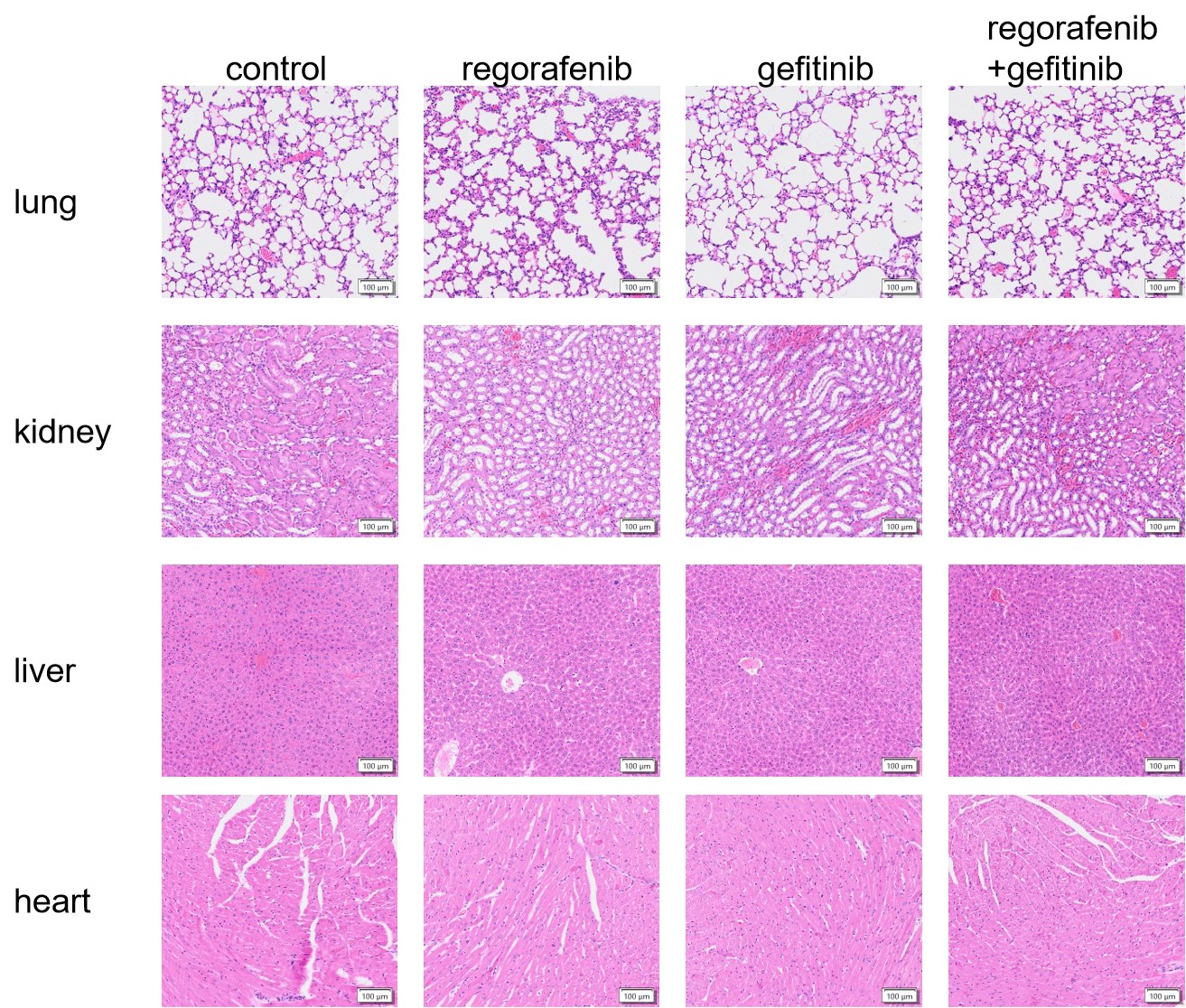

Supplement: SUPPLEMENTARY FIGURE 1 — HE staining of lung, kidney, liver and heart. [file Image_1.JPEG]
